# A novel approach for exploring climatic factors limiting current pest distributions: A case study of *Bemisia tabaci* in north-west Europe and assessment of potential future establishment in the United Kingdom under climate change

**DOI:** 10.1371/journal.pone.0221057

**Published:** 2019-08-27

**Authors:** Catherine D. Bradshaw, Deborah Hemming, Richard Baker, Matthew Everatt, Dominic Eyre, Anastasia Korycinska

**Affiliations:** 1 Met Office Hadley Centre, Exeter, United Kingdom; 2 Birmingham Institute of Forest Research, School of Geography, Earth and Environmental Sciences, Edgbaston, Birmingham, United Kingdom; 3 Plant Health Risk and Horizon Scanning Team, Defra, Sand Hutton, York, United Kingdom; Agricultural Research Organization Volcani Center, ISRAEL

## Abstract

*Bemisia tabaci* (the tobacco whitefly) is an important agricultural pest of global significance primarily because of its ability to transmit multiple damaging plant viruses. To date, UK outbreaks of the whitefly have been restricted to glasshouses and there are no records of the whitefly establishing outdoors during the summer. This is despite the fact that annual degree-day models (that estimate accumulated warmth over the year above the development threshold), indicate that *B*. *tabaci* has the thermal potential for multiple summer generations in the UK. A set of 49 climate indices calculated using the present day climate (1986–2015) were therefore compared between the UK and the south of France, where *B*. *tabaci* is able to establish outdoors, to identify the factors limiting its establishment. The number of cold days and nights in summer, as well as the time spent within the whitefly’s optimum temperature range, were most significantly different between the two areas. These indices may impact the development of *B*. *tabaci* and offer an explanation for the absence of the whitefly outdoors in the UK during the summer. Further analyses undertaken with climate projections suggest that in a 2–4°C warmer world this pest could pose a risk to outdoor UK crops in July and August. A clear south-north gradient can be demonstrated for these indices. Linking any possible northwards spread of *B*. *tabaci* populations outdoors in France with changes in these indices could therefore provide an important indicator of any change in the risks of outdoor populations of this species developing in the UK. The effectiveness of climate indices in pest risk analysis is compellingly demonstrated, and it is recommended that in-depth comparisons of climatic indices between areas of pest presence and absence are conducted in other situations where forecasting the risks of pest establishment are complex and challenging.

## Introduction

*Bemisia tabaci* (tobacco whitefly) is an important agricultural pest with a worldwide distribution, but is currently non-native to the UK. It is able to transmit upwards of 121 viruses, many of which can cause significant damage to food crops [1]. As a result, non-European populations of the whitefly are included in Annex IAI of the EU Plant Health Directive 2000/29/EC, which prohibits their introduction and spread within EU member states, and requires that their host plants are subject to targeted monitoring by plant health inspectors at the border. The UK also has a Protected Zone for European populations of the whitefly, and is therefore subject to a heightened level of surveillance and management against *B*. *tabaci*.

The Committee on Climate Change Adaptation Sub-Committee [2] has identified new and emerging animal and plant pests and diseases as one of the principal climate change risks and key research priorities for the UK. This is because future climate change may allow pests and pathogens to survive better over winter in the UK and extend their current range, and once a pest or pathogen has established, they become difficult to eradicate [2]. Risk management strategies therefore rely on early warning systems and surveillance to identify these pests and pathogens as soon as possible.

*Bemisia tabaci* has been defined as a complex of a possible 28 morphologically indistinguishable “cryptic” species, however, it has been demonstrated that there is even greater complexity [3–4]. *Bemisia tabaci* consists of a sibling species group and haplotypes based on mitochondrial DNA and some of the well-known haplotypes, MEAM1 (Middle East-Asia Minor 1 species) and MED (Mediterranean species), showing moderate to high levels of genetic diversity. The two species (or biotypes) of concern found in France are:

1. *B*. *tabaci* MEAM1; also recorded in the literature as: Biotype B and *B*. *argentifolii*.
2. *B*. *tabaci* MED; also recorded in the literature as: Biotype Q and the poinsettia whitefly.

In China and the Mediterranean region, including southern France, MED is more widespread and is displacing MEAM1, probably because it has greater resistance to insecticides [5–6]. In the UK, *B*. *tabaci* findings are not regularly identified to biotype, but it has been noted that both MEAM1 and MED *Bemisia* biotypes are frequently intercepted at the border, especially on poinsettia cuttings [7].

**Fig 1:**
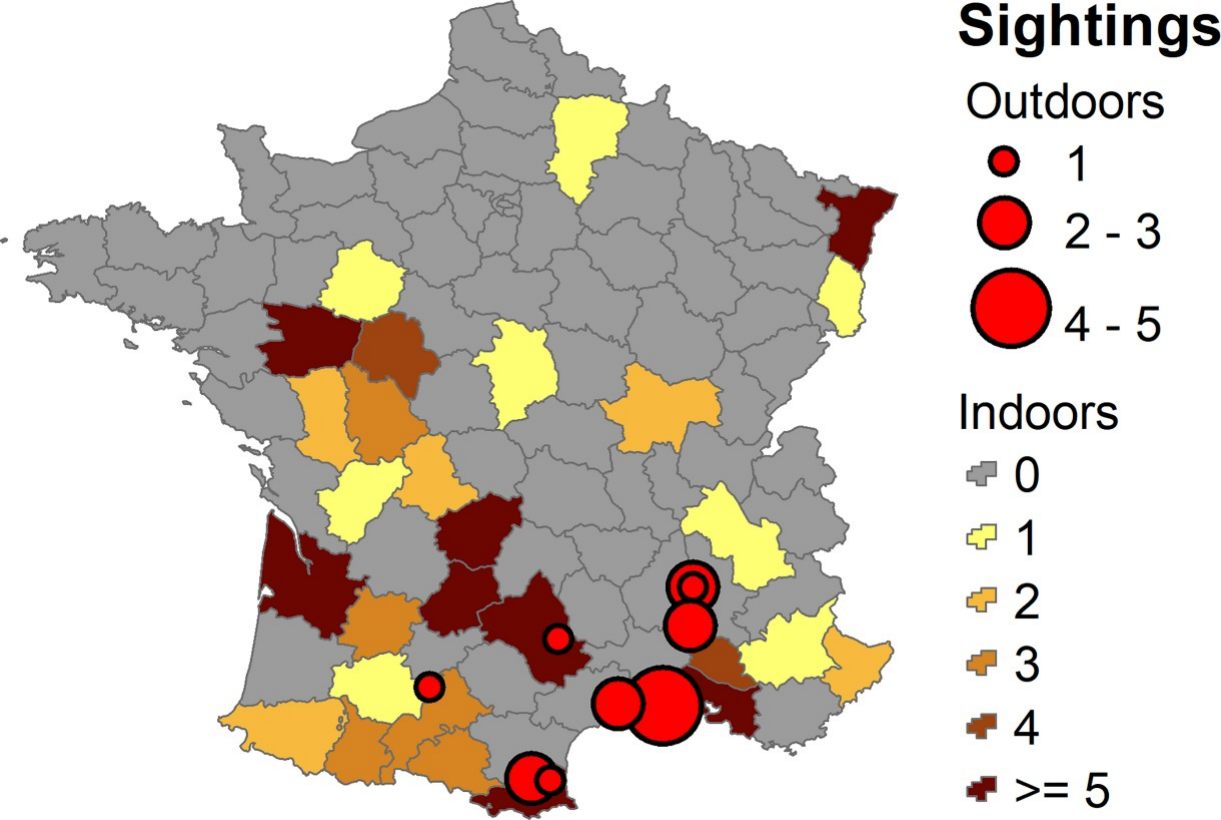
Recorded occurrences of indoor and outdoor *B*. *tabaci* in France. The larger the red circle, the more records from that site (point locations) and the darker the colour of the region, the more records from that départment. They were collected from 1996–2016 by the National Plant Protection Organisation and various technical institutes. Data courtesy of Phillipe Reynaud at ANSES, the French Agency for Food, Environmental and Occupational Health & Safety.

In 2017 *B*. *tabaci* was identified in 448 samples sent in by inspectors from England and Wales (including interceptions at airports, etc.) (Fera Science Ltd., unpublished data). Any findings of this pest in the UK are subject to eradication by the destruction of infested plants and/or a treatment programme. However, eradication is not straightforward and from 1998–2015 there were between 7 and 35 outbreaks per year [8]. Currently, these outbreaks are restricted to glasshouses, with no records of establishment outdoors during the summer, despite the opportunity for *B*. *tabaci* to escape from glasshouses. The records of the whitefly from neighbouring France are also predominantly from glasshouses, except for a small number of records in the south (Fig 1). This is despite the opportunity for *B*. *tabaci* to escape from glasshouses and move onto plants outdoors. *Bemisia tabaci* can feed on more than 800 plants species [9], many of which are present in the UK and France during the summer. Broccoli, cauliflower, squash and carrot, for example, are grown outdoors in both countries and have been shown to be some of the most preferred hosts for *B*. *tabaci* in China [10].

To explain the inability of *B*. *tabaci* to establish outdoors in the UK and the north of France during the summer, various hypotheses were tested. One explanation is that the whitefly does not experience enough accumulated warmth to complete its life cycle. Accumulated degree-day models can be used to determine whether there are sufficient degree-days above a minimum threshold for the development of the insect to complete its lifecycle [11]. Outputs of these models were mapped to show the locations where the whitefly could complete its lifecycle and the number of generations that could be completed. Alternatively, a second explanation is that other climate indices, such as climate variability and temperature extremes, may impact the whitefly, and these climate indices were compared between the UK and southern France.

To understand how the values of these climatic indices will alter under climate change, and to identify potential changes in the risk of future outbreaks of *B*. tabaci, these indices were projected forward based on rises of 1.5, 2 and 4°C above pre-industrial levels (1861–1880).

## Materials and methods

### *Bemisia tabaci* taxonomy and known temperature thresholds

Since *Bemisia tabaci* has been defined as a complex of morphologically indistinguishable “cryptic” species [3], a detailed study of the literature was undertaken to select the most appropriate temperature thresholds. Temperature thresholds extracted from the literature are given in Table 1. Temperature responses vary, not only according to each *B*. *tabaci* biotype, but also depend on the host plant and even the variety, as summarised by [1]. For example, “the developmental time of *B*. *tabaci* recorded at 25°C ranges from 17.3 to 22.8 days when reared on either aubergine, tomato, sweet potato, cucumber, bean or pepper” [12].

**Table 1:** Key temperature thresholds for *B*. *tabaci*.

Biological property	MEAM1 (Biotype B)	MED (Biotype Q)	Other	Reference
Min temperature for development	8.7°C			[13]
	11.5–16°C			[14]
	9.87°C			[1]
	9.67°C			[15]
	15°C			[16]
		8–14°C		[17]
		10.2°C		[12]
		11.11°C		[1]
Max temperature for development/survival	37.5–41.2°C			[13]
	43–44°C	43–44°C		[16]
		32–35°C		[17]
Day-degrees for development (base temperature)	401–437 (8.7°C)			[13]
	307 (11.5°C)			[14]
	345 (9.67°C)			[15]
		327 (8°C)		[17]
		400 (10.2°C)		[12]
Optimal temperature range for development/survival	32°C			[13]
	26°C			[14]
	20–30°C	20–30°C		[16]
		32.5°C		[12]
			25–27°C	[18]
Min temperature for adult flight			19.2–22.0°C	[19]
			6–8°C	[20]
Min temperature for oviposition		14°C		[17]

Much of the extensive literature on *B*. *tabaci* was published before it was known to be a complex and so only temperature thresholds that can be clearly related to MEAM1 or MED have been selected. Some exceptions have been included in the “Other” column where the paper refers to MEAM1 and MED without distinguishing between them, or the reference is unique in the *B*. *tabaci* literature because it includes parameters such as adult flight.

A large number of papers provide evidence of *B*. *tabaci*’s response to cold temperatures but these are too anecdotal to be used predictively. However, [21] showed experimentally that a MED population from southern France survived for four days and four nights with temperatures cycling between –5°C and 15°C, similar to those occurring in our winter. Recent literature from eastern Asia has also explored the role of maximum temperatures of short and long duration on survival [16]. There are no records of variables other than temperature being important for the development and survival of *B*. *tabaci*, but it is known from other species that relative humidity in combination with temperature can be important, especially at low temperatures [22].

### Theoretical *B*. *tabaci* development outdoors

In order to determine the theoretical mean annual number of generations possible for *B*. *tabaci* in the UK and France, a degree-day model was used based on a modified formula [11]. This formula was slightly amended to account for the situation where the daily mean temperature equals the development threshold. The degree-day model was run with daily maximum and minimum temperatures interpolated to a 25 km grid obtained from JRC-MARS climate datasets for 1986–2015 (see next section). The higher of the two MED degree-day parameters was chosen, i.e. the 10.2°C and 400 degree days [12], in order to depict the UK as less favourable for development. The ideal approach is to use data from populations close to the study areas. However, MEAM1 is an invasive species, and as the studies are some years old, the present composition of the populations in southern France is unknown. Therefore, the decision was taken to use data for the MEAM1 species as a whole, and investigate the range of reported values with two different scenarios. The first scenario depicts the UK as most favourable (8.7°C and 404 accumulated degree days) [13]. We chose 404 from the range cited in Table 1 because data collected for the PRATIQUE project give 8.7°C and 404 accumulated degree days for the egg-adult development. The second scenario depicts the UK as least favourable (11.5°C and 307 accumulated degree days) [14]. These two scenarios illustrate the potential variation in published work. These three models were run and the predicted mean number of generations that could be completed successfully in a year was calculated and mapped.

### Climate indices

Climate indices are a way of characterizing the more extreme aspects of climate and climate variability. In order to characterize the climate of the region where *B*. tabaci has been found outdoors, all 27 indices defined by the Expert Team on Climate Change Detection and Indices [23] were used, together with additional relevant indices defined by the European Climate Assessment & Dataset [24].

The first step in our approach was to determine an appropriate gridded daily dataset upon which to carry out the quantitative comparison. The use of station data was ruled out because of the localised nature of the data and difficulties in identifying truly comparable stations. Two datasets are available that cover the UK and France at a resolution of ~25km: E-Obs [25] and JRC-MARS [26], as described in the next section.

As well as the standard set of indices, additional pest-relevant indices were developed which include specific biological thresholds and functions. These were identified through expert knowledge from Defra’s Plant Health Risk and Horizon Scanning Team, and are detailed in Table 2. All indices have been calculated for each month of each year and the results are presented as the mean index value across the 30-year climatology period.

**Table 2:** Biology-related temperature thresholds.

Parameter	Threshold	Justification	Index Code	Reference
Mean temperature	15°C	Minimum temperature at which full development from egg to adult is possible	TGge15	[16]
Mean temperature AND Maximum temperature	20°C (mean) / 30°C (max)	Range outside which survival is substantially reduced	TGge20_TXle30	[16]

Refer to Table A in S1 Appendix for the full index definitions.

### Climate data

The E-Obs data are routinely used in the assessment of model forecasts at the Met Office (e.g. [27]), whereas the JRC-MARS data are routinely used by Defra for pest risk analyses (e.g. [28]).

#### E-Obs

E-Obs was developed for the purpose of regional climate model evaluation. E-Obs version 14.0 provides daily gridded precipitation, temperature and sea level pressure data for land areas only, covering the period January 1950 to August 2016. The data are generated by interpolating monthly values using a thin-plate spline method and then interpolating the daily anomalies of the 11348 stations’ data using a global kriging method. Station coverage is denser in the UK than France for both precipitation and temperature. Annual average standard error for mean temperature across all of Europe over the period of this present study is ~0.69°C for years prior to 2000, rising to over 0.7°C subsequently due to a marked drop off in the number of stations [25].

#### JRC-MARS

JRC-MARS (Joint Research Center-Monitoring Agricultural ResourceS) data was developed by AGRI4CAST to provide crop production information to the European Commission [29]. For this reason, the data are designed to be representative depictions of the weather in a typical agricultural holding within each grid cell, and therefore the median altitude of the non-irrigated arable land within the grid cell is chosen. JRC-MARS version 1.0 provides daily gridded temperature, wind speed, vapour pressure, precipitation, potential evaporation and evapotranspiration, radiation and snow depth data for land areas only, covering the period 1975 to present. Most of the data are generated by calculating the average of the daily data from up to the four most suitable weather stations for each grid cell from the ~9450 total stations; precipitation is taken directly from the single most suitable station identified. Suitable stations are identified according to a similarity score based on the distance, altitude difference, distance to coast and distance to uniform weather regions between the station and the grid cell centre. For the 7 weather stations most similar to the grid cell centre, a set score for all possible combinations of 1 up to 4 of these weather stations is determined, and the highest scoring set used in the interpolation. For temperature and vapour pressure parameters, the data are then corrected for altitude differences between the station and the grid cell centre using a moist adiabatic lapse rate of -0.006°Cm^-1^ and -2.5% per 100m increase [30].

#### E-Obs and JRC-MARS dataset comparison

For the purpose of the present study, the JRC-MARS data were translated onto the same grid as the E-Obs data by using the nearest grid cell. As the two datasets are generated using different methodologies, a simple student’s t-test comparison between the two was performed (where the variables were provided for both datasets). This comparison identified that there were some months where the maximum of the daily minimum temperatures, the mean of the daily minimum temperatures, the maximum of the daily mean temperature, the maximum of the daily precipitation amounts and sum of the daily total precipitation were statistically different between E-Obs and JRC-MARS. For this reason, all of the analyses conducted in this study use both datasets to enable common features to be assessed for their relevance to pest survival. Data are taken for the period 1986–2015.

#### Climate change projections

The data used for future climate projections comes from the HELIX project [31–32]. The high resolution climate model used was HadGEM3; the Met Office’s high resolution global climate model [33–34]. To reduce computation time, the model was run in atmosphere-only mode rather than fully coupled atmosphere-ocean mode. This was achieved by using fixed sea surface temperatures (SSTs) and sea-ice concentrations (SICs) from different driving models run at a lower resolution as part of the Coupled Model Intercomparison Project Phase 5 (CMIP-5; [35]) initiative. In order to incorporate the uncertainty from the climate projections, a total of 6 of the 35 CMIP-5 models were used to drive HadGEM3 (Table 3), chosen in the HELIX project as representative of the full model projections spread. The climate model data has been bias-corrected relative to the Princeton Global Forcings V2 reanalysis data product [36] according to the ISI-MIP bias correction protocol [37]. The CMIP-5 models were forced with the Representative Concentration Pathway 8.5 greenhouse gas emissions scenario from 1979–2100. This scenario is consistent with ongoing high emissions and hence features a rapid rate of warming.

**Table 3:** The timings of passing global warming levels (GWL) 1.5°C, 2°C and 4°C.

SST Driving Model^a^	GWL 1.5	GWL2	GWL4
ACCESS1-0	2026 (2017–2036)	2040 (2031–2050)	2081 (2072–2091)
GFDL-ESM2M^b^	2036 (2027–2046)	2051 (2042–2061)	-
IPSL-CM5A-LR	2024 (2015–2034)	2035 (2026–2045)	2071 (2062–2081)
IPSL-CM5A-MR	2023 (2014–2033)	2036 (2027–2046)	2069 (2060–2079)
MIROC-ESM-CHEM	2020 (2011–2030)	2032 (2023–2042)	2068 (2059–2078)

Central years of the range are given for the high resolution HadGEM3 simulations and the 20 year range used for the analysis (in brackets). It is assumed that the observed warming from the pre-industrial period to the reference period (1861–1880 mean to 1981–2010 mean) is 0.61°C [38].

^a^ Only five ensemble members of the full six-member HadGEM3 ensemble available were used in this analysis as the HadGEM3 model driven by HadGEM2-ES sea surface temperatures and sea-ice concentrations exhibited anomalous behaviour in the daily precipitation data after the bias correction process.

^b^ The GFDL-ESM2M model did not reach an increase of 4°C above the pre-industrial climate before 2100 and therefore it was not possible to analyse the HadGEM3-GFDL-ESM2M ensemble member at GWL4.

The future time periods considered are identified by Global Warming Levels (GWLs)—identified as mitigation targets in the Paris Agreement—which are set at a global annual mean rise of 1.5, 2 and 4°C relative to the pre-industrial climate (1861–1880). The central year of GWL1.5, GWL2 and GWL4 for each ensemble member is defined as the year in which the 20-year running mean of the global annual mean temperature anomaly first passes the specific temperatures of 1.5, 2, and 4°C respectively. The temperature anomaly is calculated relative to the pre-industrial period: defined as the mean of 1861–1880. The bias-corrected central GWL years are calculated assuming the observed warming from the pre-industrial period to the bias correction reference period (1861–1880 mean to 1981–2010 mean) is 0.61°C [38]. In accordance with the HELIX protocol, 20 years of data either side of the central year were used to define the climatology for each GWL (Table 3). For consistency with the E-Obs and JRC data, HadGEM3 data is also taken for the baseline period 1986–2015.

### Case study regions

**Fig 2:**
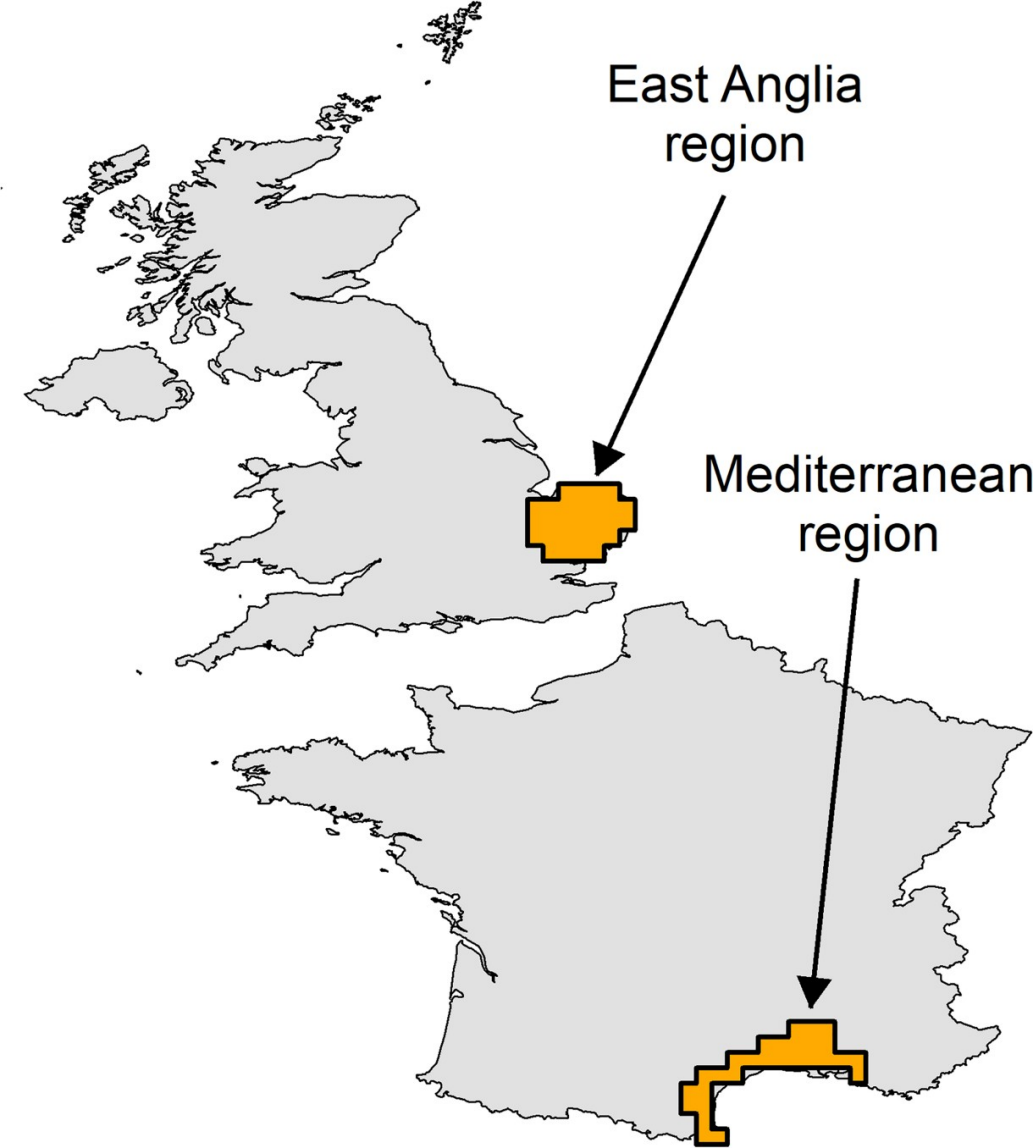
Location of East Anglia (UK) and the Mediterranean (France) regions as defined in this study.

**Fig 3:**
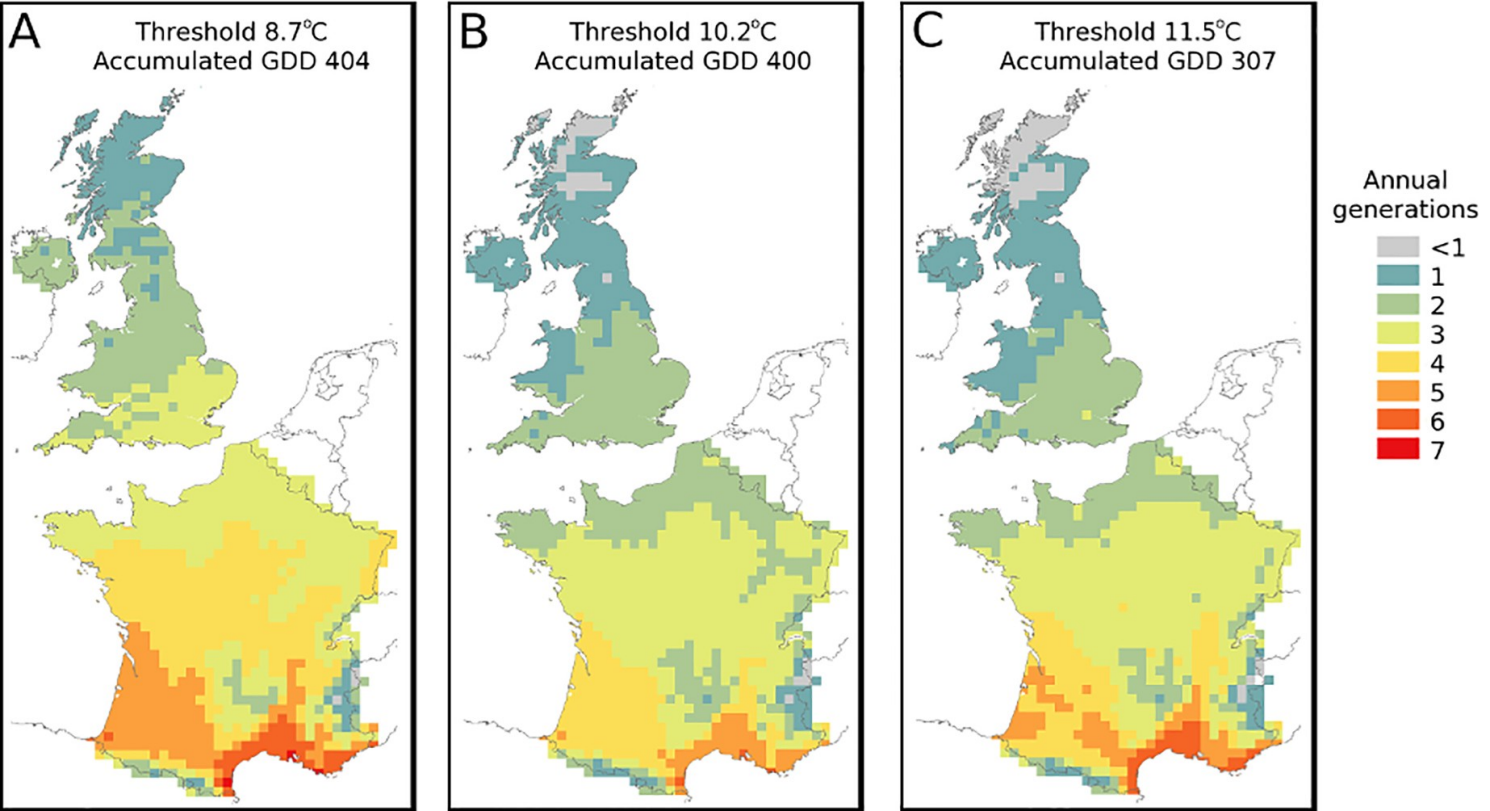
Theoretical mean number of generations using data for 1986–2015. Based on A: 8.7°C as the minimum threshold for development and 404 as the accumulated number of degree-days to complete a generation based on [13] for MEAM1, B: 10.2°C as the minimum threshold for development and 400 as the accumulated number of day-degrees to complete a generation based on [12] for MED (Biotype Q) and C: 11.5°C as the minimum threshold for development and 307 as the accumulated number of day-degrees to complete a generation based on [14] for MEAM1 (Biotype B).

East Anglia was chosen as an example region in the UK (Fig 2), in which 2–3 generations of *B*. *tabaci* are expected according to the annual degree-day model (Fig 3). Climate indices were used to characterise aspects of the climate most different to that of Mediterranean France.

## Results

### Annual degree-day models results under current climatic conditions

Fig 3B and 3C show that, on average, MEAM1 and MED should have enough accumulated day-degrees in the present climate (1986–2015) to complete one generation over England, Wales and much of Scotland. Two generations are predicted in much of southern and central England. Only in parts of the Scottish Highlands and associated Islands are there areas which are unsuitable for even one generation to be completed. Whereas the alternative parameters for MEAM1 [13], shown in Fig 3A, indicate that one generation could occur everywhere, with at least 3 generations being possible in southern and central England. The thresholds used for MED were the higher of the values reported (refer to Table 1). Mapping these means the UK was depicted as less favourable than the other set of thresholds would imply. Given these less favourable data still show that much of the UK is theoretically suitable for 1–2 generations per year, this increased our confidence that at least some parts of the UK would be suitable for outdoor establishment. Choosing the lower threshold from [17] would make the UK appear more favourable still.

However, it should be noted that many summers over the 30-year period represented by these data will have been much hotter (or cooler) than the mean, potentially allowing more (or fewer) generations to have developed from any whiteflies escaping from glasshouses. Moreover, there will be numerous locations within each 25 km grid cell where temperatures accumulate faster (or slower) than the value representing each cell that is based on the estimated mean agricultural altitude [29].

In France, the number of potential mean annual generations increases up to a maximum of 7 along the Mediterranean coast. Except for areas of high ground (The Massif Central in central southern France, the Alps in the east and the Pyrenees to the south), there is a general southward increase in generation number.

### Climate indices under current climatic conditions

Although the climate indices were found to be significantly different in all months, the results show the summer months (June, July, and August) to be the most significantly different. These are also the key months for potential *B*. *tabaci* development outdoors. The indices showing the most significant differences between East Anglia and Mediterranean France are TG10p and TN10p: the number of cold days and cold nights that are colder than the 10^th^ percentile of days and nights in Mediterranean France (refer to Table A in S1 Appendix for the full index definitions).

The JRC-MARS and E-Obs datasets do show significant differences for some of the climate indices used in this study, however, the indices showing most significant differences between East Anglia and Mediterranean France are consistent between the two datasets (Table B in S1 Appendix). This provides evidence that the spatial and temporal differences observed in these indices represent genuine climate differences, rather than variations caused by the specific dataset used.

**Fig 4:**
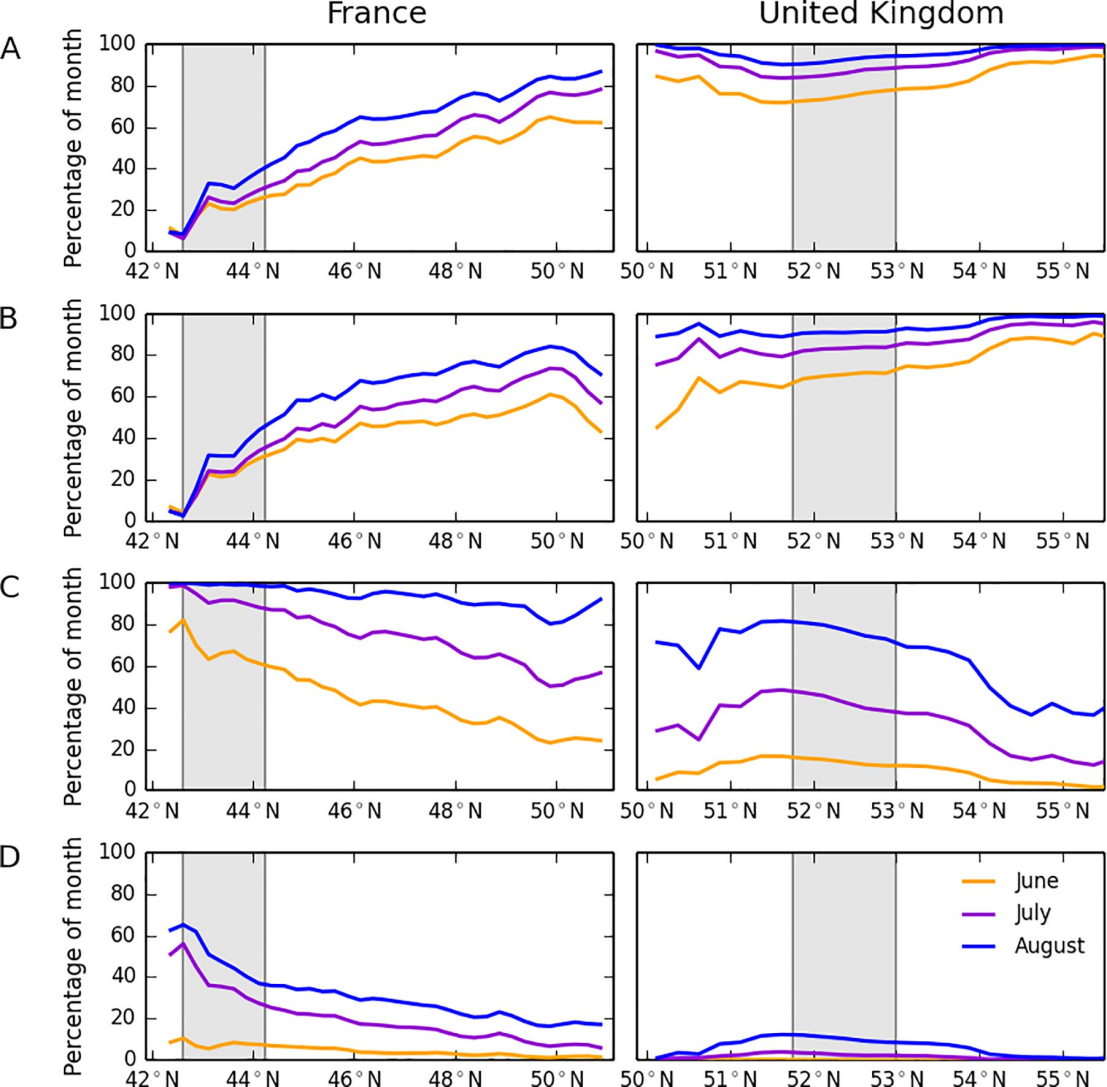
Latitudinal transects from the EObs dataset. A: Percentage of summer months as cold as the 10^th^ percentile cold days in Mediterranean France, B: Percentage of month as cold as the 10^th^ percentile cold nights in Mediterranean France, C: Percentage of month available for optimal development conditions from egg to adult (>15°C), D: Percentage of month available for optimal survival conditions (> = 20°C AND < = 30°C). The left-hand panels show the transects through France and the right-hand panels show the transects through the United Kingdom. The vertical shaded bars correspond to the regions for Mediterranean France and East Anglia as shown in Fig 2. Latitudinal averages are constructed by excluding the Central Massif, the Alps, the Pyrenees, Wales, Northern Ireland and Scotland from the datasets as high elevations are likely to hinder whitefly colonisation. The indices for the number of days in the month meeting each condition were converted to percentage of the month by dividing by the number of days in each month and multiplying by 100.

Fig 4A and 4B clearly show that there is a strong gradient across France in the number of days as cold as the coldest 10% of days and nights in Mediterranean France, but quite a flat profile across the UK. The indices incorporating user-defined thresholds, although not the most significantly different between regions, are informative. Mid- to southern France spends more than 80% of the summer months in conditions optimal for *B*. *tabaci* development (temperature during egg to adult > 15°C), but there is a sharp decline in the time spent in optimal conditions for development across northern France and into the UK from south to north (Fig 4C). Similarly, mid- and southern France experience the greatest amount of time in conditions optimal for survival (temperature > = 20°C and < = 30°C), but the amount of time diminishes in northern France and into the UK (Fig 4D). The UK experiences only a very small amount of time in conditions optimal for *B*. *tabaci* survival (<15% of time during the summer months). However, the results also show that, even in Mediterranean France, conditions are only optimal for *B*. *tabaci* survival for 40–60% of the time during these warmest months. This may contribute to the low number of outdoor observations as compared to Crete where outdoor populations of whitefly are a very serious problem [39]. The corresponding values for Crete—amount of time in conditions optimal for survival (temperature > = 20°C and < = 30°C)—using the same methodology as for the UK and France, is 70% of the time during June-August for the whole island.

### Climate change projections

**Fig 5:**
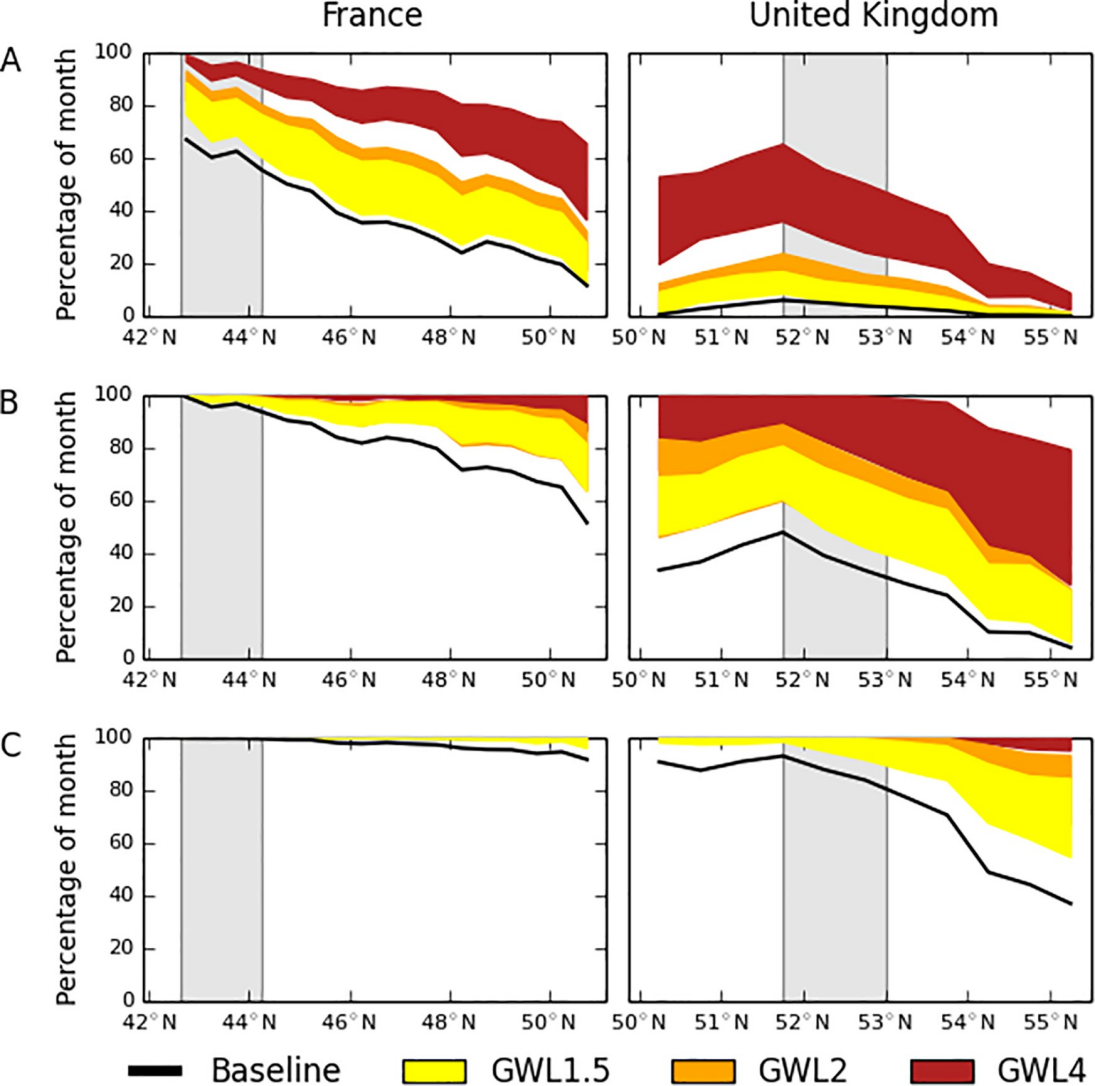
**Latitudinal transects showing the percentage of month (A: June, B: July, C: August) available for optimal development conditions from egg to adult (>15°C) under the three global climate warming levels (GWL) of 1.5, 2 and 4°C above preindustrial conditions.** The left-hand panels show the transects through France and the right-hand panels show the transects through the United Kingdom. The vertical shaded bars correspond to the regions for Mediterranean France and East Anglia as shown in Fig 2. Latitudinal averages are constructed by excluding the Central Massif, the Alps, the Pyrenees, Wales, Northern Ireland and Scotland from the datasets as high elevations are likely to hinder whitefly colonisation. The indices for the number of days in the month meeting each condition were converted to percentage of the month by dividing by the number of days in each month and multiplying by 100.

**Fig 6:**
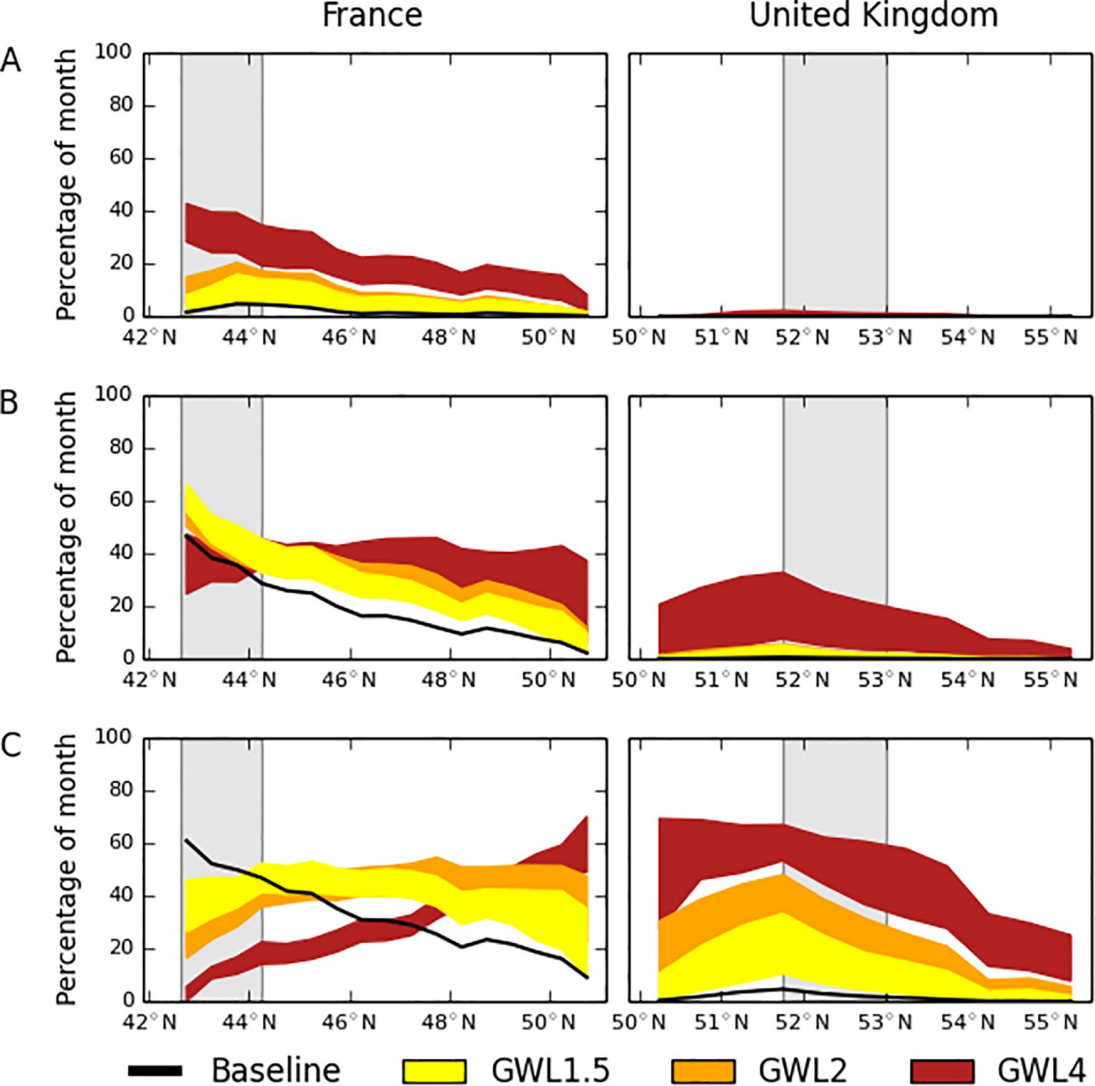
**Latitudinal transect showing the percentage of month (A: June, B: July, C: August) available for optimal survival conditions (> = 20°C AND < = 30°C) under the three global climate warming levels (GWL) of 1.5, 2 and 4°C above preindustrial conditions.** The left-hand panels show the transects through France and the right-hand panels show the transects through the United Kingdom. The vertical shaded bars correspond to the regions for Mediterranean France and East Anglia as shown in Fig 2. Latitudinal averages are constructed by excluding the Central Massif, the Alps, the Pyrenees, Wales, Northern Ireland and Scotland from the datasets as high elevations are likely to hinder whitefly colonisation. The indices for the number of days in the month meeting each condition were converted to percentage of the month by dividing by the number of days in each month and multiplying by 100.

As the climate warms by GWL2 in July, and by GWL1.5 in August, the amount of time with optimal development conditions for *B*. *tabaci* in East Anglia becomes statistically indistinguishable to that of Mediterranean France in the current climate (Fig 5 compared to Fig 4C; see also Table C and Fig A in S1 Appendix). Similarly, the amount of time with optimal survival conditions for *B*. *tabaci* in East Anglia becomes statistically indistinguishable in July and August between GWL1.5 and GWL2 (Fig 6; see also Fig B in S1 Appendix). However, by GWL4 the difference for July and August reverses and Mediterranean France rises above the optimal survival temperature for *B*. *tabaci* (Fig 6). The climate indices results indicate that there would be a gradual northward shift across France in the locations most suitable for *B*. *tabaci* development and survival around GWL2 and then a shift across into the UK by GWL4.

The number of cold days and nights as cold as the 10^th^ percentile of days and nights in Mediterranean France under current climate conditions for the Mediterranean France region remains significantly different to East Anglia under the climate projections (Table B in S1 Appendix). However, the comparison shows that some parts of southern UK will be comparable to parts of south-western France where *B*. *tabaci* has been found outdoors at GWL4 (Figs C-F in S1 Appendix).

## Discussion

### Limitations for establishment

The annual degree-day models showed that *B*. *tabaci* could theoretically complete one generation across most of Scotland, 1–3 generations over England and Wales, and 3–4 generations over northern France (Fig 3) under current climate conditions. Accumulated warmth is therefore not currently the limiting factor for the establishment of the whitefly in the UK and northern France. When climate indices of East Anglia in the UK were compared with Mediterranean France, however, significant differences were shown that could impact on the establishment of the whitefly. All four of the indices relate to the time under which the whitefly experiences cool temperatures (or temperatures outside of its preferred temperature zone).

As a poikilothermic ectotherm, *B*. *tabaci* is unable to regulate its own body temperature and is influenced by environmental temperature, and is therefore susceptible to chilling injury as a result of low temperature exposure [40]. Chilling injury often involves the reduction and loss of membrane, organelle and enzyme function, and other processes, which in turn reduce the survival and performance of sub-lethal characteristics of the organism concerned [41–43].

The temperatures experienced during the cold days and nights relative to Mediterranean France may be low enough to cause chilling injury to *B*. *tabaci*, thereby inhibiting development and preventing establishment in the UK as compared to the south of France. It is unlikely, therefore, that this pest will establish outdoors in the UK under current climate conditions. The number of consecutive cold days in summer is also greater in the UK than in the south of France (~20 days per months for the UK as compared to <5 days per month for southern France; see Fig G in S1 Appendix). This presents a further risk, as chilling injury is more likely to occur the longer an invertebrate is exposed to low temperatures [44].

### Impacts of climate change

Although *B*. *tabaci* is unlikely to become established outdoors in the UK under current climate conditions, with continued future climate change, the risk of establishment outdoors in the UK is expected to increase. The movement of pests northward in France with climate change has already been demonstrated for the pine processionary moth (*Thaumetopoea pityocampa*) [45], highlighting the increasing pest threat to the UK.

Taken together, the results of the future climate indices analyses indicate that a 2°C global temperature rise above pre-industrial levels will reduce the prevalence of *B*. *tabaci* outdoors in Mediterranean France in July and August as conditions become sub-optimal for survival. The rise in temperature could potentially lead to a greater threat to the UK in the months of June and September and/or a shift northwards in distribution (up to and including southern UK) as conditions in the UK become more favourable. These results differ from previous studies that suggest a northward spread for *B*. *tabaci* across France as a result of future climate change and an increase in its range throughout southern Europe but not northern Europe [1,15]. However, the Gilioli study ([15]) used a physiologically based population dynamics model to map the potential distribution of *B*. *tabaci* in Europe. The population of immatures and adults were constrained with a temperature dependent mortality rate function linked to a development rate function. Although the model outputs correspond closely to the known limits of *B*. *tabaci* distribution outdoors in Europe, by linking mortality to the suitability for development they did not take account of any other factors, such as the influence of cold weather. Furthermore, the climate data used was limited to monthly mean minimum and maximum temperatures with an interpolation routine based on geographical location and the time of sunrise used to derive hourly temperature data. Unlike the current study, which used a suite of climate model projections, the climate change scenario tested was flat (1 and 2°C were added to each hourly temperature).

Our study suggests that *B*. *tabaci* could establish outdoors in East Anglia and across southern England in the future, which would make eradication of outbreaks in future more challenging than the current situation, where outbreaks are confined to glasshouses. Given the crucial need for an early warning system against *B*. tabaci, we have identified that key indices for *B*. *tabaci* survival and development in summer change gradually with increasing latitude (northwards from areas of France with a Mediterranean climate). This means that monitoring any possible northwards spread of *B*. *tabaci* populations outdoors in France should also provide an important indicator of any change in the risks of outdoor populations of this species developing in the UK.

### Further applications

This study has focused on the application of climate indices for assessing the risk of establishment of *B*. *tabaci* in northern France and the UK. Such an in-depth comparison of climatic indices between areas of pest presence and absence outdoors to identify additional factors that may be responsible for limiting the potential area of pest distribution is almost unique in the history of pest risk analysis. It was made possible by a strong collaboration between teams of experts in climatology and pest risk analysis utilising continental-wide high-resolution climate datasets. The technique has a much wider application for using climate indices and high resolution daily data to model any pests in any country where traditional degree-day models do not match the observed situation. It is therefore recommended that such methods are adopted in other situations where forecasting the risks of pest establishment are complex and challenging.

In addition to helping clarify the limitations of phenology models, this work will provide valuable evidence for more complex species distribution models, such as CLIMEX (see: http://climatemodel.net/climex.htm), which include stress indices based on species responses to cold, hot, wet and dry conditions. CLIMEX generates an ecoclimatic index that represents the year-round suitability for establishment. Factors, such as cold stress, are parameterised to reflect the likelihood of overwintering survival rather than the extent to which cool periods in summer can also limit the area of establishment. In addition to parameterisation based on published climatic responses, a key advantage of CLIMEX is the ability to infer parameters from the distribution of a pest. For species such as *B*. *tabaci*, which is a complex of 28 cryptic species, the distribution of each member of the complex is poorly known making it difficult to apply this CLIMEX attribute. In this situation, phenology models, such as the ones used in this project, based primarily on climate response thresholds, are of greater utility.

## Supporting information

S1 Appendix **Table A. Definition of the climate indices used**.
**Table B. Student’s t-test t-statistics for the most significantly different climate indices between the East Anglia and Mediterranean France regions in the UK and France respectively.** Also shown are the t-statistics for the biology-related indices (Table 2). Indices included are significantly different at the 99% confident interval in a Student’s t-test. Refer to Table A for the description of the indices.
**Table C. Student’s t-test t-statistics for the most significantly different climate indices between the East Anglia and Mediterranean France regions, in the UK and France respectively, in the baseline period and the biology-related indices (refer to Table 1 and Table 2 for more details).** Indices included are significantly different at the 99% confident interval by the number of models shown in brackets. Refer to Table A for the description of the indices.* Note that only 4 of the 5 models reach GWL4 by 2100.
**Fig A. Spatial distribution of percentage of month available for optimal development conditions from egg to adult (>15°C) for the current climate observations, the climate model baseline and under the three global climate warming levels (GWL) of 1.5, 2 and 4°C above preindustrial conditions**.
**Fig B. Spatial distribution of percentage of month available for optimal survival conditions (> = 20°C AND < = 30°C) for the current climate observations, the climate model baseline and under the three global climate warming levels (GWL) of 1.5, 2 and 4°C above preindustrial conditions**.
**Fig C. Latitudinal transect showing the percentage of month (A: June, B: July, C: August) as cold as the 10**^**th**^ **percentile cold days in Mediterranean France (under current climate conditions in the Mediterranean France region) under the three global climate warming levels (GWL) of 1.5, 2 and 4°C above preindustrial conditions.** The left-hand panels show the transects through France and the right-hand panels show the transects through the United Kingdom. The vertical shaded bars correspond to the regions for Mediterranean France and East Anglia as shown in Fig 2. Latitudinal averages are constructed by excluding the Central Massif, the Alps, the Pyrenees, Wales, Northern Ireland and Scotland from the datasets as high elevations are likely to hinder whitefly colonisation.
**Fig D. Spatial distribution of percentage of month as cold as the 10**^**th**^ **percentile cold days in Mediterranean France (under current climate conditions in the Mediterranean France region) for the current climate observations, the climate model baseline and under the three global climate warming levels (GWL) of 1.5, 2 and 4°C above preindustrial conditions**.
**Fig E. Latitudinal transect showing the percentage of month (A: June, B: July, C: August) as cold as the 10**^**th**^ **percentile cold nights in Mediterranean France (under current climate conditions in the Mediterranean France region) under the three global climate warming levels (GWL) of 1.5, 2 and 4°C above preindustrial conditions.** The left-hand panels show the transects through France and the right-hand panels show the transects through the United Kingdom. The vertical shaded bars correspond to the regions for Mediterranean France and East Anglia as shown in Fig 2. Latitudinal averages are constructed by excluding the Central Massif, the Alps, the Pyrenees, Wales, Northern Ireland and Scotland from the datasets as high elevations are likely to hinder whitefly colonisation.
**Fig F. Spatial distribution of percentage of month as cold as the 10**^**th**^ **percentile cold nights in Mediterranean France (under current climate conditions in the Mediterranean France region) for the current climate observations, the climate model baseline and under the three global climate warming levels (GWL) of 1.5, 2 and 4°C above preindustrial conditions**.
**Fig G. Spatial distribution of the number of consecutive days as cold as the 10**^**th**^ **percentile cold days in Mediterranean France (under current climate conditions in the Mediterranean France region) for the EObs data for June, July and August**.
(PDF)

S1 Data (XLSX)
