# Supplementary material for: A novel approach for exploring climatic factors limiting current pest distributions: A case study of Bemisia tabaci in north-west Europe and assessment of potential future establishment in the United Kingdom under climate change
Source: PLoS One. 2019 Aug 27;14(8):e0221057. doi: 10.1371/journal.pone.0221057 (PMC6711539; doi:10.1371/journal.pone.0221057)
Supplement: S1 Appendix — Table A. Definition of the climate indices used. Table B. Student’s t-test t-statistics for the most significantly different climate indices between the East Anglia and Mediterranean France regions in the UK and France respectively. Also shown are the t-statistics for the biology-related indices (Table 2). Indices included are significantly different at the 99% confident interval in a Student’s t-test. Refer to Table A for the description of the indices. Table C. Student’s t-test t-statistics for the most significantly different climate indices between the East Anglia and Mediterranean France regions, in the UK and France respectively, in the baseline period and the biology-related indices (refer to Table 1 and Table 2 for more details). Indices included are significantly different at the 99% confident interval by the number of models shown in brackets. Refer to Table A for the description of the indices.* Note that only 4 of the 5 models reach GWL4 by 2100. Fig A. Spatial distribution of percentage of month available for optimal development conditions from egg to adult (>15°C) for the current climate observations, the climate model baseline and under the three global climate warming levels (GWL) of 1.5, 2 and 4°C above preindustrial conditions. Fig B. Spatial distribution of percentage of month available for optimal survival conditions (> = 20°C AND < = 30°C) for the current climate observations, the climate model baseline and under the three global climate warming levels (GWL) of 1.5, 2 and 4°C above preindustrial conditions. Fig C. Latitudinal transect showing the percentage of month (A: June, B: July, C: August) as cold as the 10th percentile cold days in Mediterranean France (under current climate conditions in the Mediterranean France region) under the three global climate warming levels (GWL) of 1.5, 2 and 4°C above preindustrial conditions. The left-hand panels show the transects through France and the right-hand panels show the transects through the United [file pone.0221057.s001.pdf]

Table A. Definition of the climate indices used.

Index Code	Index Name	Index Method
TG10p	Cold days	$$TG10p_j = \left(\sum_{i=1}^{I} days\right) \mid TG_{ij} < TG_{in}10$$ Where, $TG_{ij} = Mean\ daily\ temperature\ for\ day\ i\ of\ period\ j$ $TG_{in}10 = Calendar\ day\ 10th\ percentile\ mean\ temperature\ for\ a\ 5\ day\ centred\ on\ each\ calendar\ day\ in\ the\ baseline\ climatology$
TN10p	Cold nights	$$TN10p_j = \left(\sum_{i=1}^{I} days\right) \mid TN_{ij} < TN_{in}10$$ Where, $TN_{ij} = Minimum\ daily\ temperature\ for\ day\ i\ of\ period\ j$ $TN_{in}10 = Calendar\ day\ 10th\ percentile\ minimum\ temperature\ for\ a\ 5\ day\ window\ centred\ on\ each\ calendar\ day\ in\ the\ baseline\ climatology$
TGge15	Number of days with mean temperature greater than or equal to 15°C	$$TGge_j = \sum_{i=1}^{I} days \mid (TG_{ij} \geq 15\ ℃)$$ Where, $TG_{ij} = Mean\ daily\ temperature\ for\ day\ i\ of\ period\ j$
TGge20_TXle30	Number of days with mean temperature greater than or equal to 20°C AND max temperature less than or equal to 30°C	$$TGge\_TXle_j = \sum_{i=1}^{I} days \mid (TG_{ij} \geq 20\ ℃\ AND\ TX_{ij} \leq 30\ ℃)$$ Where, $TG_{ij} = Mean\ daily\ temperature\ for\ day\ i\ of\ period\ j$ $TX_{ij} = Max\ daily\ temperature\ for\ day\ i\ of\ period\ j$

Table B. Student's t-test t-statistics for the most significantly different climate indices between the East Anglia and Mediterranean France regions in the UK and France respectively. Also shown are the t-statistics for the biology-related indices (Table 2). Indices included are significantly different at the 99% confident interval in a Student's t-test. Refer to Table A for the description of the indices.

Index	Month	E-Obs	JRC-MARS
TG10p	June	33.2	39.1
	July	32.7	37.0
	August	35.5	36.1
TN10p	June	30.4	32.3
	July	31.8	33.5
	August	31.9	32.5
TGge15	June	-19.9	-20.1
	July	-7.3	-7.6
	August	-7.0	-7.2
TGge20_TXle30	June	-20.4	-23.1
	July	-10.9	-10.4
	August	-12.6	-12.3

Table C. Student's t-test t-statistics for the most significantly different climate indices between the East Anglia and Mediterranean France regions, in the UK and France respectively, in the baseline period and the biology-related indices (refer to Table 1 and Table 2 for more details). Indices included are significantly different at the 99% confident interval by the number of models shown in brackets. Refer to Table A for the description of the indices.* Note that only 4 of the 5 models reach GWL4 by 2100.

Index	Month	HadGEM3 (Baseline)	HadGEM3 (GWL1.5)	HadGEM3 (GWL2)	HadGEM3 (GWL4)*
TG10p	June	21.4 to 37.3 (5)	16.2 to 57.2 (5)	20.1 to 41.0 (5)	7.0 to 22.0 (4)
	July	26.7 to 40.1 (5)	26.4 to 65.6 (5)	21.3 to 58.2 (5)	6.4 to 15.7 (4)
	August	23.3 to 39.0 (5)	36.7 to 58.7 (5)	29.0 to 46.3 (5)	5.6 to 18.7 (4)
TN10p	June	18.6 to 28.5 (5)	11.5 to 31.5 (5)	13.4 to 28.0 (5)	6.2 to 18.2 (4)
	July	25.3 to 33.1 (5)	16.6 to 49.1 (5)	13.1 to 36.1 (5)	6.9 to 12.8 (4)
	August	25.1 to 43.3 (5)	20.1 to 33.7 (5)	17.0 to 36.8 (5)	9.3 to 14.9 (4)
TGge15	June	-10.9 to -14.5 (5)	-3.5 to -10.3 (5)	-3.5 to -9.7 (5)	-3.8 to -5.3 (2)
	July	-2.8 to -4.1 (4)	-3.9 (1)	**Not significantly different**	**Not significantly different**
	August	-3.5 to -4.3 (5)	**Not significantly different**	**Not significantly different**	**Not significantly different**
TGge20_TXle30	June	-13.4 to -15.4 (5)	-12.1 to -17.3 (5)	-8.5 to -14.2 (5)	-4.1 to -7.8 (3)
	July	-10.5 to -14.4 (5)	-6.9 (1)	2.9 to 4.0 (2)	7.5 to 15.6 (4)
	August	-11.5 to -15.9 (5)	-2.8 to -5.2 (3)	**Not significantly different**	8.0 to 17.6 (4)

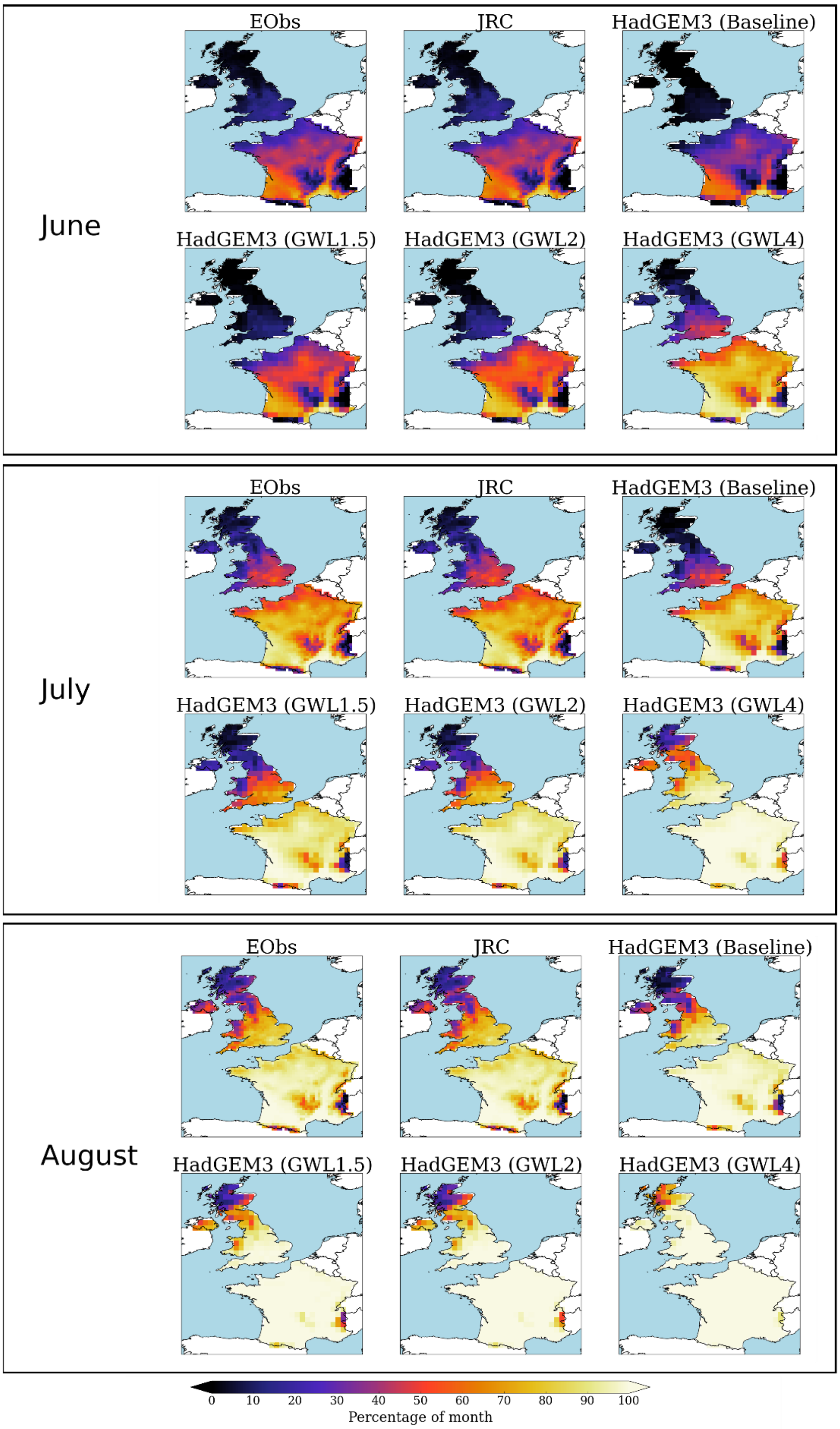

Fig A. Spatial distribution of percentage of month available for optimal development conditions from egg to adult (>15°C) for the current climate observations, the climate model baseline and under the three global climate warming levels (GWL) of 1.5, 2 and 4°C above preindustrial conditions.

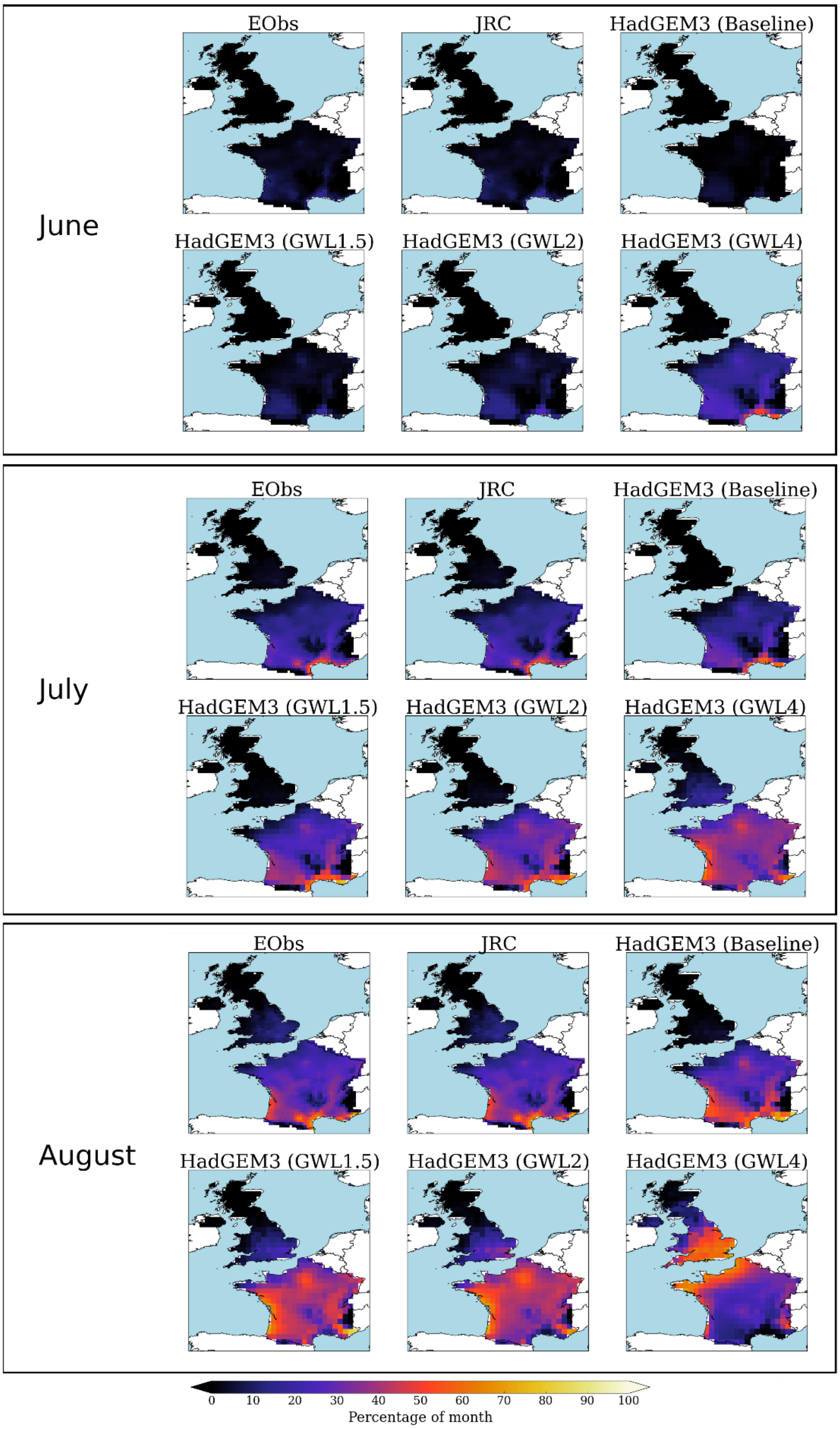

Fig B. Spatial distribution of percentage of month available for optimal survival conditions (>=20°C AND <=30°C) for the current climate observations, the climate model baseline and under the three global climate warming levels (GWL) of 1.5, 2 and 4°C above preindustrial conditions.

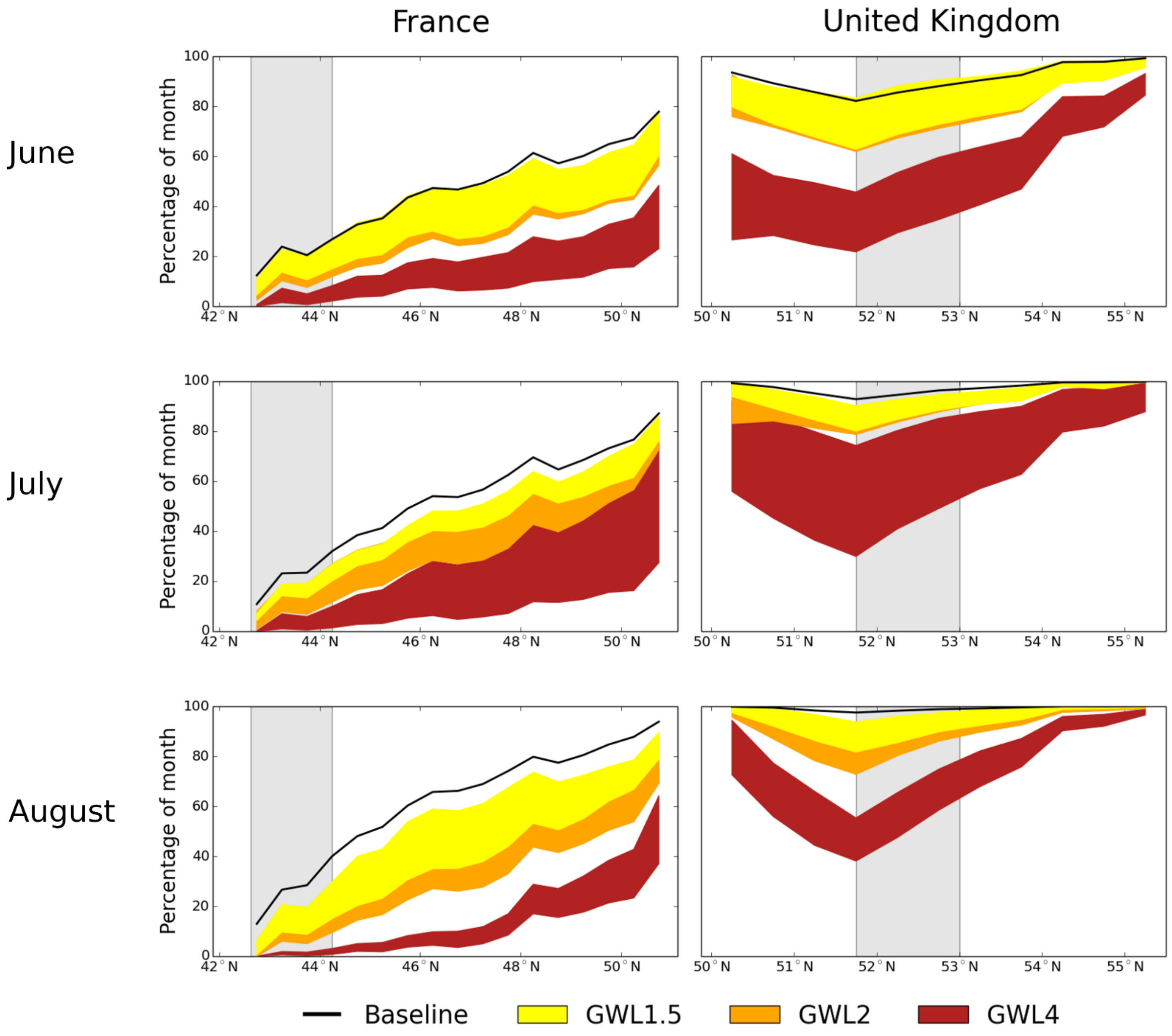

Fig C. Latitudinal transect showing the percentage of month (A: June, B: July, C: August) as cold as the 10th percentile cold days in Mediterranean France (under current climate conditions in the Mediterranean France region) under the three global climate warming levels (GWL) of 1.5, 2 and 4°C above preindustrial conditions. The left-hand panels show the transects through France and the right-hand panels show the transects through the United Kingdom. The vertical shaded bars correspond to the regions for Mediterranean France and East Anglia as shown in Figure 2. Latitudinal averages are constructed by excluding the Central Massif, the Alps, the Pyrenees, Wales, Northern Ireland and Scotland from the datasets as high elevations are likely to hinder whitefly colonisation.

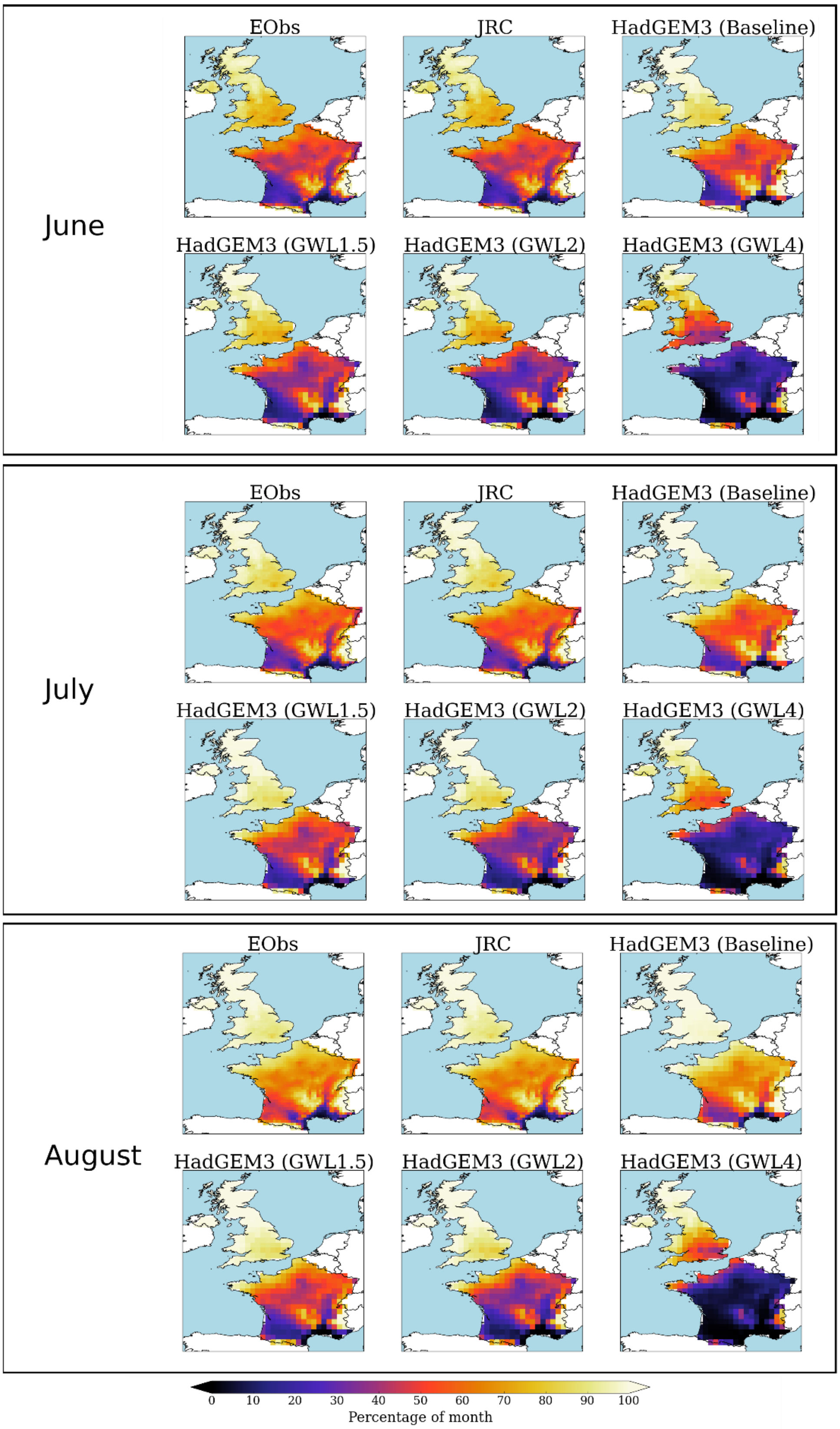

Fig D. Spatial distribution of percentage of month as cold as the 10th percentile cold days in Mediterranean France (under current climate conditions in the Mediterranean France region) for the current climate observations, the climate model baseline and under the three global climate warming levels (GWL) of 1.5, 2 and 4°C above preindustrial conditions.

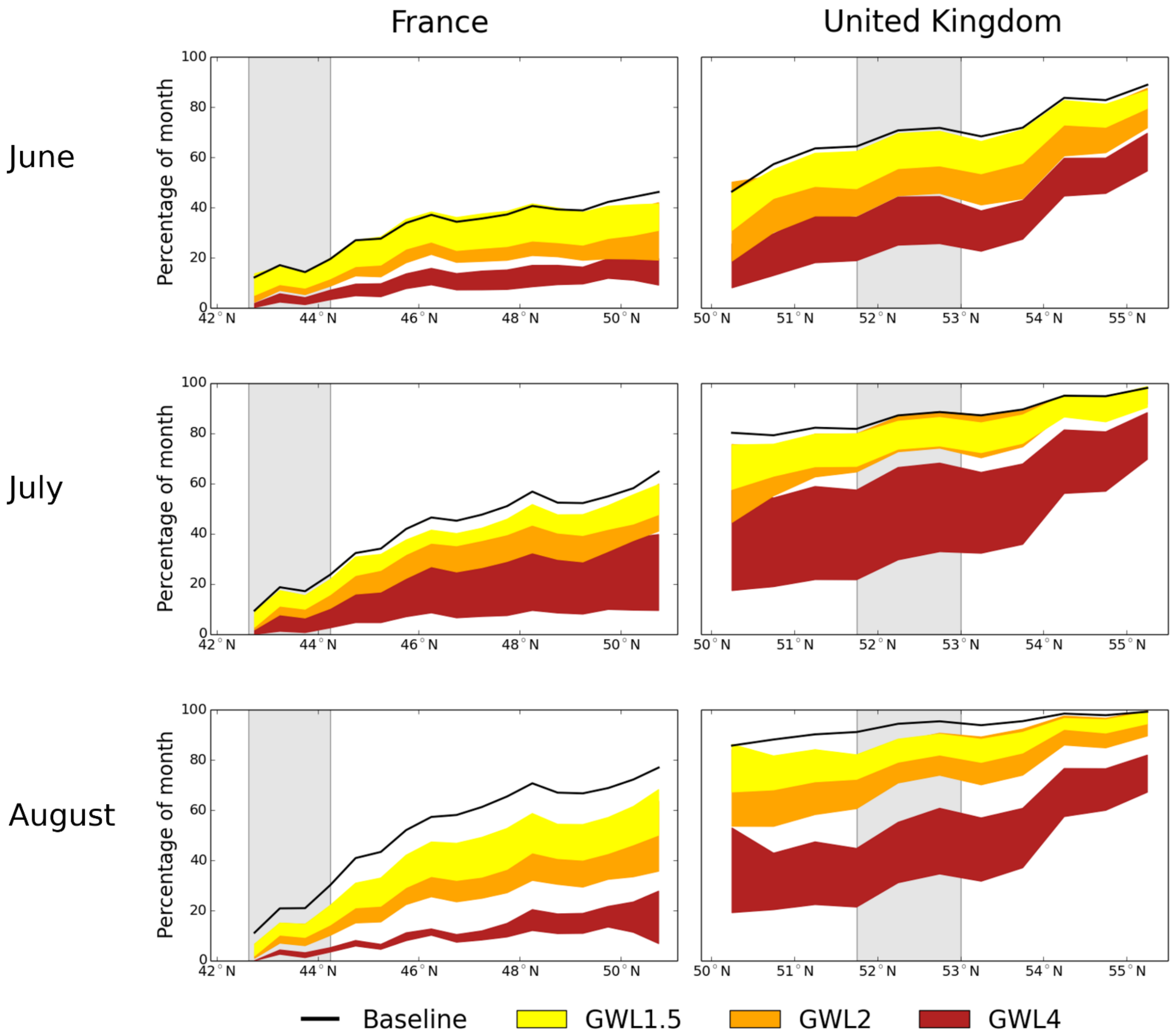

Fig E. Latitudinal transect showing the percentage of month (A: June, B: July, C: August) as cold as the 10th percentile cold nights in Mediterranean France (under current climate conditions in the Mediterranean France region) under the three global climate warming levels (GWL) of 1.5, 2 and 4°C above preindustrial conditions. The left-hand panels show the transects through France and the right-hand panels show the transects through the United Kingdom. The vertical shaded bars correspond to the regions for Mediterranean France and East Anglia as shown in Figure 2. Latitudinal averages are constructed by excluding the Central Massif, the Alps, the Pyrenees, Wales, Northern Ireland and Scotland from the datasets as high elevations are likely to hinder whitefly colonisation.

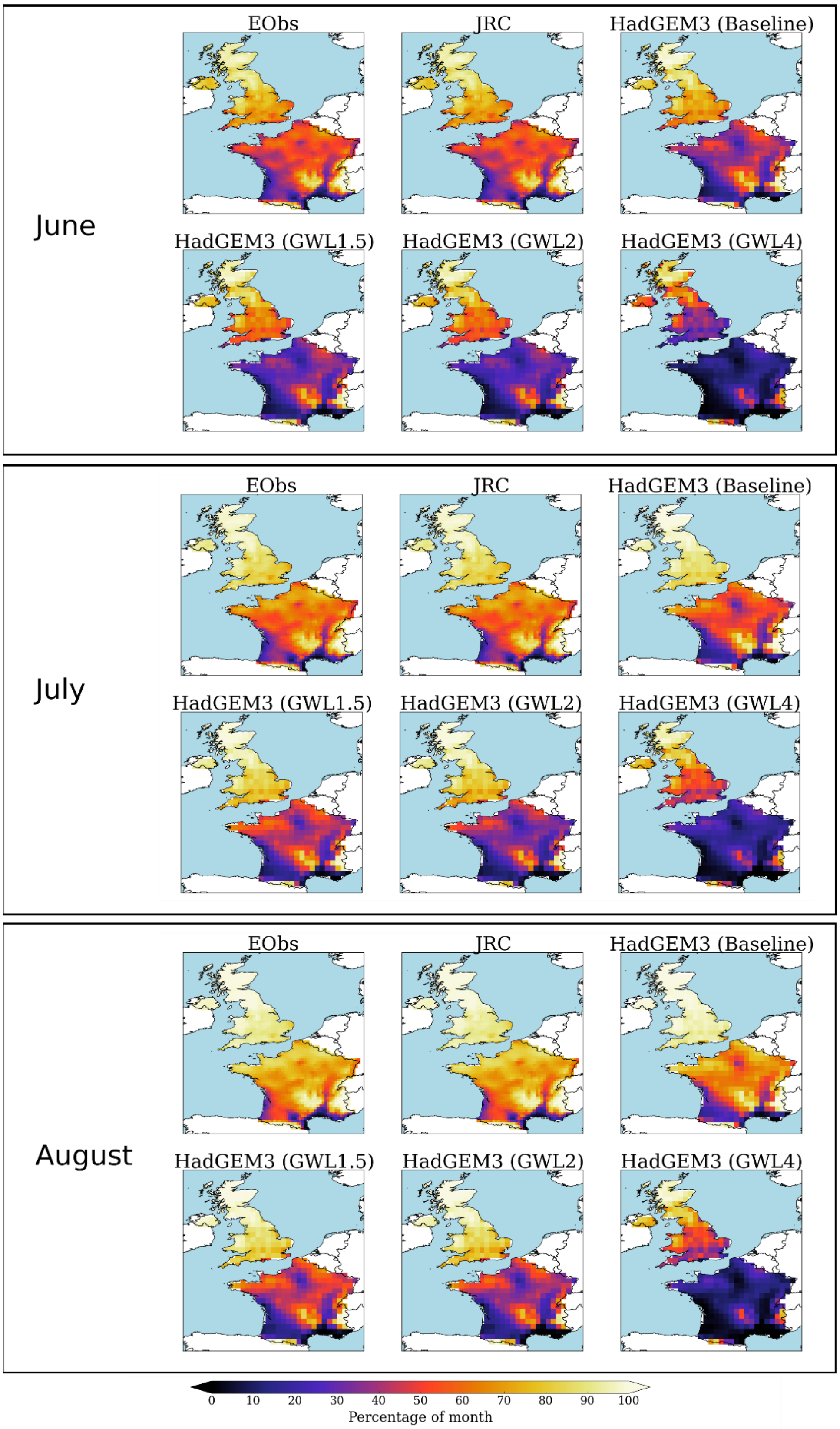

Fig F. Spatial distribution of percentage of month as cold as the 10th percentile cold nights in Mediterranean France (under current climate conditions in the Mediterranean France region) for the current climate observations, the climate model baseline and under the three global climate warming levels (GWL) of 1.5, 2 and 4°C above preindustrial conditions.

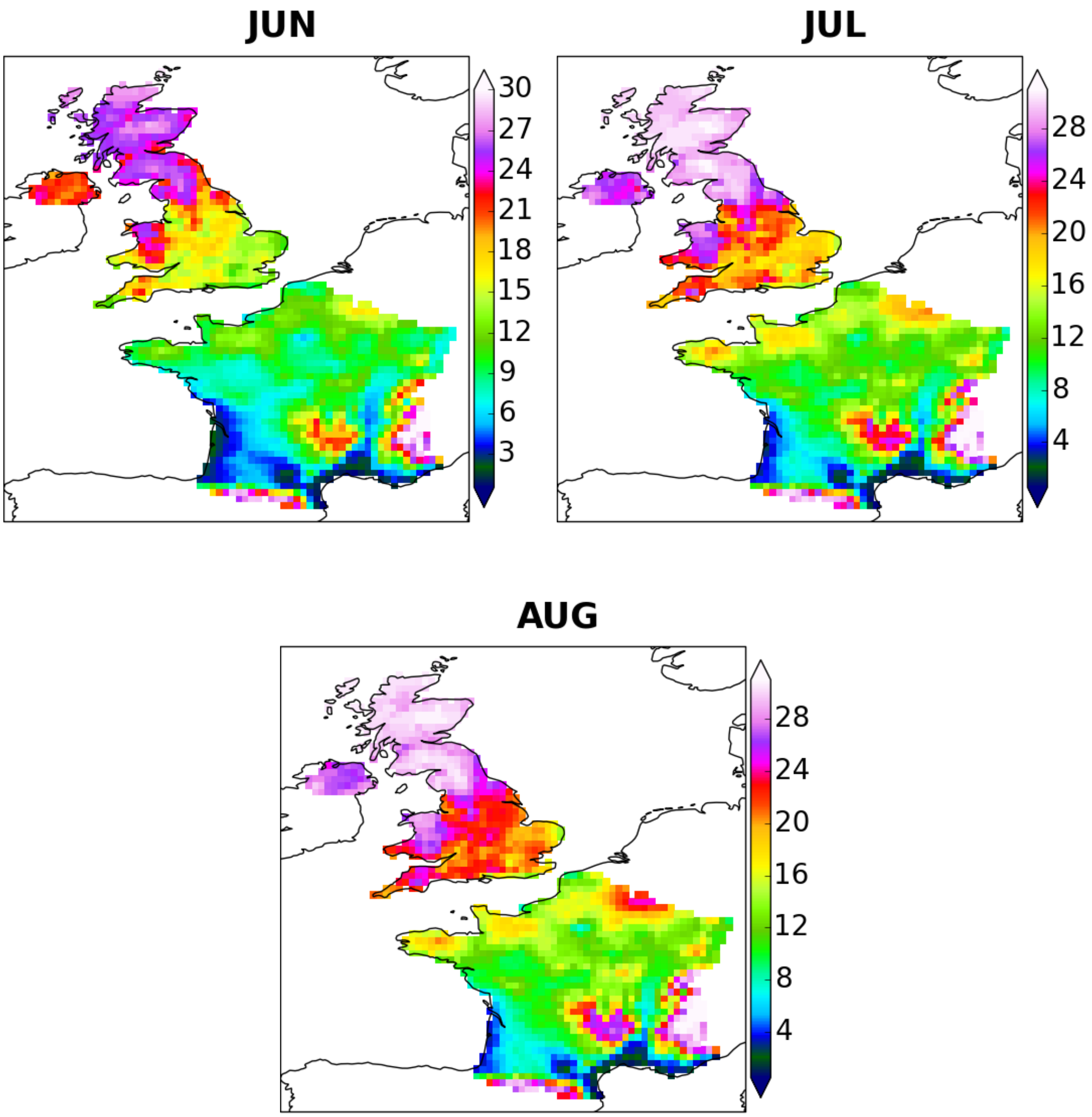

Fig G. Spatial distribution of the number of consecutive days as cold as the 10th percentile cold days in Mediterranean France (under current climate conditions in the Mediterranean France region) for the EObs data for June, July and August.
